# Changing clinical manifestations of Gaucher disease in Taiwan

**DOI:** 10.1186/s13023-023-02895-z

**Published:** 2023-09-15

**Authors:** Wen-Li Lu, Yin-Hsiu Chien, Fuu-Jen Tsai, Wuh-Liang Hwu, Yen-Yin Chou, Shao-Yin Chu, Meng-Ju Li, An-Ju Lee, Chao-Chuan Liao, Chung-Hsing Wang, Ni-Chung Lee

**Affiliations:** 1 Department of Clinical Pathology, Chi Mei Medical Center, Tainan, Taiwan; 2 Department of Medical Genetics, National Taiwan University Hospital, 8 Chung-Shan South Road, Taipei, 10041 Taiwan; 3 Department of Pediatrics, National Taiwan University Hospital, Taipei, Taiwan; 4 Division of Medical Genetics, Pediatric Endocrinology and Metabolism, China Medical University Children’s Hospital, 2, Yude Road, North District, Taichung City, 40447 Taiwan; 5 School of Chinese Medicine, College of Medicine, China Medical University, Taichung, Taiwan; 6 Department of Pediatrics, National Cheng Kung University Hospital, College of Medicine, National Cheng Kung University, Tainan, Taiwan; 7 Department of Pediatrics, Buddhist Tzu Chi General Hospital, Hualien, Taiwan; 8 School of Medicine, Tzu Chi University, Hualien, Taiwan; 9 Department of Pediatrics, National Taiwan University Hospital Hsin-Chu Branch, Hsinchu City, Taiwan; 10 School of Medicine, College of Medicine, China Medical University, Taichung, Taiwan; 11 Center for Precision Medicine, China Medical University Hospital, Taichung, Taiwan

**Keywords:** Gaucher disease, Enzyme replacement therapy, Phenotype, Newborn screening

## Abstract

**Background:**

Gaucher disease (GD) is a lysosomal storage disorder characterized by deficient glucocerebrosidase activity that results from biallelic mutations in the *GBA1* gene. Its phenotypic variability allows GD to be classified into 3 subtypes based on the presence and extent of neurological manifestations. Enzyme replacement therapy (ERT) has been available for all patients with GD in Taiwan since 1998. Newborn screening (NBS) for GD has been available since 2015. This study attempted to unveil the clinical features of patients diagnosed with GD during different eras in Taiwan.

**Materials and methods:**

Data from the health records of two tertiary hospitals responsible for two-thirds of the patients with GD in Taiwan were used. The study population included all patients identified as having GD between 1998, and April 2022, in these two hospitals for review. A total of 42 individuals were included, six of whom were diagnosed by NBS.

**Results:**

Our cohort presented a higher proportion of GD3 individuals, both by clinical suspicion and by NBS diagnosis, than that reported worldwide. The major subtypes that were recognized following NBS diagnosis were GD2 and GD3. The majority of GD patients carry at least one p.Leu483Pro variant. The 5-year survival rates were 0% for GD2 patients and 100% for patients with other subtypes. Patients diagnosed during the post-NBS era were free of symptoms on initial presentation, except for those with the GD2 subtype. For those diagnosed earlier, ERT was shown to be effective in terms of improved hemograms and prevented bone crises. However, the neurological symptoms in GD3 patients progressed despite ERT intervention.

**Conclusion:**

ERT is essential in reversing the hematological presentations and preventing the skeletal complications of GD. Timely diagnosis of GD with NBS allows for early intervention with ERT to prevent disease progression and complications. However, the need for effective intervention for neurological dysfunction remains unmet.

**Supplementary Information:**

The online version contains supplementary material available at 10.1186/s13023-023-02895-z.

## Background

Among the more than 70 types of lysosomal storage disorders (LSDs) identified to date, Gaucher disease (GD; MIM 230800, 230900, and 231000 for types 1, 2, and 3, respectively) is one of the most common types [1–3]. It is an autosomal recessive disorder of protein misfolding as well as glycolipid storage characterized by a deficiency in the lysosomal enzyme activity of glucocerebrosidase, also known as acid-ß-glucosidase, due to biallelic mutations in the *GBA1* gene [4]. Of the more than 860 *GBA1* mutations known to be associated with GD [5], four mutations—NM_000157.4:c.1226A > G (p.Asn409Ser, aka. N370S), c.1448T > C (p.Leu483Pro, aka. L444P), c.84dup (p.Leu29fs, aka. 84GG), and c.115 + 1G > A (aka. IVS2 + 1)—are responsible for the majority of the disease population: 80–90% of all mutations are present in Ashkenazi Jewish patients, and 50–60% are present in non-Ashkenazi Jewish patients [6]. In contrast, two mutations—c.1448T > C (p.Leu483Pro) and the triply mutant c.1448T > C, c.1483T > G, and c.1497G > C (*RecNci*I)—were found to be more prevalent in Taiwan [7]. Such mutations disrupt the breakdown of glucocerebroside into glucose and ceremide, leading to glucocerebroside accumulation within the lysosomes of macrophages, which brings about the presence of Gaucher cells, or engorged macrophages, in organs and bone marrow, resulting in the clinical manifestations of GD. Intriguingly, phenotypic variability among patients with the same Gaucher genotype explains the different disease manifestations or responses to therapies, and vice versa.

Given its phenotypic heterogeneity, GD is categorized into three clinical subtypes based on the extent of neurological manifestations—nonneuronopathic type 1 (GD1), acute neuronopathic type 2 (GD2), and chronic neuronopathic type 3 (GD3)[8, 9]. The majority of patients are diagnosed with GD1, which is a chronic disorder that lacks neurological involvement and presents mainly with hematological, visceral, and skeletal manifestations [10–14]. In addition to the manifestations experienced by GD1 patients, GD2 and GD3 patients suffer from neurological involvement of earlier and later onset, respectively. Several attempts had been made to differentiate GD2 and GD3 by the time of onset and the rate of progression, although the neurological features overlap in neuropathic GD (nGD) [9]. In general, GD2 manifests and deteriorates rapidly before 6 months of age, resulting in death typically within the first triennium of life [14]. The presentation of GD3 is subacute or chronic yet clinically heterogeneous; many patients with mild cognitive involvement and a well-controlled seizure disorder live full and productive lives, others suffer from developmental delay and seizure, and still others suffer from cognitive impairment and dementia [9, 10, 14]. However, the intelligence performance is heterogenous as well considering that those without cognitive involvement exhibit IQs in the normal to high range [15]. In the era of NBS, the symptomatology and patient prognosis at a very young age is unclear. In addition to the hematological, visceral, and neurological manifestations, patients with GD may display symptoms and signs of skeletal disorders, pulmonary disorders, neuropsychiatric disorders, metabolic disorders, immunologic disorders, malignancies/cancer, cardiovascular disorders, and gastrointestinal disorders [16]. Nonetheless, potential drug–drug interactions with any concomitant medications used to treat these comorbidities should be considered.

Current treatment options for GD comprise enzyme replacement therapy (ERT), which replenishes deficient glucocerebrosidase with a recombinant enzyme, and substrate reduction therapy (SRT), which reduces the upstream synthesis of glucocerebroside. While SRT is approved for the treatment of GD1 and ERT is approved for the treatment of GD1 and GD3, many who suffer from this condition may not be able to afford such a treatment due to the rarity of the disease [17–21]. Thanks to the Rare Disease and Orphan Drug Act, people with Gaucher disease have equal access to proper treatment under the coverage of the National Health Insurance in Taiwan without facing economic hardships. Since the introduction of ERT in 1998 and SRT in 2005 in Taiwan [22], Gaucher patients have been able to mitigate the influence of the disease with regular treatment. With the advent of newborn screening (NBS) for GD in 2015, the clinical features of GD have evolved in Taiwan [23]. By 30 April 2022, a total of 42 patients, including 8 patients who died before 31 December 2021, were diagnosed with GD in Taiwan [24]. Of these, 27 (64%) patients, including 4 deceased patients, were under the care of two medical centers between 1998 and April 2022. This study attempted to reveal differences with the joint data of the two hospitals, which provide care for nearly two-thirds of the Gaucher population in Taiwan.

## Materials and methods

### Patients

This was a retrospective cohort study of GD patients followed in two main tertiary hospitals in Taiwan (National Taiwan University [NTUH] and China Medical University Hospital [CMUH]), accounting for the care of 64% of the Taiwanese GD population. Patients with confirmed Gaucher disease visiting the hospital between 1998, and April 2022, were reviewed. Some of the patients had been reported [25, 26]. Medical records were retrospectively reviewed, and demographic data (age, date of birth, and sex), information pertaining to diagnosis (sonographic measurements of the liver and spleen, hemograms, age at diagnosis, molecular diagnosis, phenotype categorization, as well as presenting symptoms including skeletal manifestations, oculomotor dysfunction, poor feeding, and ichthyosis), treatment data (medical management, initial age at intervention), and data regarding evolvement under treatment (hemograms and survival status) were collected. In general, physical examination and neurological examination were done at time of diagnosis, and every 6–12 months thereafter until 2 years of age. After that, annual check would be done for registry evaluation and drug application. Patients with neurological involvement, such as gaze palsy, slow or absent saccades, intellectual disability, seizure, or intellectual impairment, were classified as having neuropathic GD following assessment by neurological examination, which evaluated mental status, posture, cranial nerves, motor systems including muscle atrophy, tone and power, sensory system, coordination, and gait, with or without electroencephalography and intelligence test via the Wechsler Intelligence Scales for Adults (WAIS) or for Children (WISC). GD2 was classified if the patient had neonatal ichthyosis or neurological symptoms before 6 months of age. Although gaze palsy is considered the required criterion for neuropathic GD [9], sometimes it is not readily perceived, especially in young patients [27]. Therefore, patients with various degrees of neurological involvement with/without oculomotor apraxia, lymphadenopathies or kyphosis were classified as having GD3.

Liver and spleen parameters included liver and spleen measurements that were converted to multiples of the age-specific uppermost limits of normal, as recommended by Konuş et al., given the substantial variations in the sizes of the liver and spleen among individuals of different ages [28].

### Statistics

Statistical analyses were conducted using IBM SPSS Statistics, versions 28.0.1.1 and 27.0.1.0, Prism 9 for macOS, versions 9.3.1 and 9.5.1, and Microsoft Excel for Mac, version 16.60. Sex, molecular diagnosis, age at diagnosis, age at symptom onset, initial presentations, age at treatment, treatment interventions, and current age/age of death were tabulated. The *p* values of the survival curve were determined by the log-rank test. All other *p* values were calculated with the Mann–Whitney test. Results with a *p* value less than 0.05 were considered statistically significant.

## Results

### Distribution of GD types

Between 1999, and April 2022, 27 patients, including 4 patients who died, were under the care of either NTUH or CMUH, as shown in Table 1. Among them, 9 patients (33%) were categorized as having GD1, 3 (11%) as having GD2, and 14 (52%) as having GD3. One case (4%) was unclassified due to the accidental death of the patient at 1 year old without other symptoms following diagnosis by newborn screening during the neonatal period. Although not statistically significant (*p* = 0.542), the incidence of GD2 increased from 5 to 33% with NBS by juxtaposing patients who were clinically diagnosed with patients who were diagnosed by NBS (Table 2).

**Table 1:** Demographics of our patients

	GD1	GD2	GD3	Unclassified
Number of cases (n = 27)	9 (33%)	3 (11%)	14 (52%)	1 (4%)
*Sex*				
Male	5 (56%)	1 (33%)	8 (57%)	1 (100%)
Female	4 (44%)	2 (67%)	6 (43%)	0 (0%)
*Molecular diagnosis*				
p.Leu483Pro/p.Leu483Pro	–	–	11 (79%)	–
p.Leu483Pro/p.Val414Leu	4 (44%)	–	–	–
p.Leu483Pro/p.Asp448His	–	–	2 (14%)	–
p.Leu483Pro/*RecNci*I^†^	–	1 (33%)	1 (7%)	–
p.Leu483Pro/p.Asn227Ser	1 (11%)	–	–	–
p.Leu483Pro/p.Asn409Ser	1 (11%)	–	–	–
p.Leu483Pro/p.Phe252Ile	1 (11%)	–	–	–
p.Leu483Pro/p.Arg170His	1 (11%)	–	–	–
p.Leu483Pro/p.Arg159Trp	–	–	–	1 (100%)
*RecNci*I/p.Asp448His	–	1 (33%)	–	–
*RecNci*I/p.Arg159Trp	1 (11%)	–	–	–
p.Phe252Val/p.328Alafs*12	–	1 (33%)	–	–
*Category of diagnosis*				
Clinical (n = 21)	8 (38%)	1 (5%)	12 (57%)	–
Newborn screening (n = 6)	1 (17%)	2 (33%)	2 (33%)	1 (17%)
*Initial presentation*				
Hepatosplenomegaly	8 (89%)	2 (67%)	12 (86%)	0 (0%)
Anemia/thrombocytopenia	7 (78%)	1 (33%)	12 (86%)	0 (0%)
Bone pain/bone crisis	3 (33%)	0 (0%)	2 (14%)	0 (0%)
Abnormal eye movements	0 (0%)	2 (67%)	1 (7%)	0 (0%)
Poor feeding	0 (0%)	2 (67%)	0 (0%)	0 (0%)
Ichthyosis	0 (0%)	1 (33%)	0 (0%)	0 (0%)
*Median [IQR] age at diagnosis, years*				
Clinical diagnosis	24.41 [3.72–35.65]	0.92	2.52 [1.87–8.70]	–
Newborn screening	0.08	0.14	0.26	0.08
*Median [IQR] age at ERT, years*				
Clinical diagnosis	29.44 [3.68–42.91]	–^‡^	3.17 [2–16.75]	–
Newborn screening	2.83	0.56	0.66	–
*Treatment*^§^				
ERT	9 (100%)	1 (33%)	14 (100%)	–
SRT	1 (11%)	0 (0%)	6 (43%)	–
Ambroxol	1 (11%)	0 (0%)	5 (36%)	–
HSCT	0 (0%)	1 (33%)	0 (0%)	–
Splenectomy	4 (44%)	0 (0%)	2 (14%)	–
5-year survival rate	100%	0%	100%	0%

IQR, interquartile range

^†^*RecNci*I: gene conversion with *GBAP*. p.Asp448His, p.Leu483Pro, p.Ala495Pro

^‡^–: no treatment

^§^Some received combined treatment with multiple drugs

**Table 2:** Comparison of the pre- and post-NBS eras

	Clinical	NBS	*p* value
Number of patients (n = 27)	21	6	
*Subtype*			
GD1	8 (38%)	1 (17%)	0.182
GD2	1 (5%)	2 (33%)	0.054
GD3	12 (57%)	2 (33%)	0.542
*Molecular diagnosis*			
p.Leu483Pro/p.Leu483Pro	10 (48%)	1 (17%)	0.182
p.Leu483Pro/p.Val414Leu	4 (19%)	–	0.256
p.Leu483Pro/p.Asp448His	2 (10%)	–	0.441
p.Leu483Pro/*RecNci*I†	–	2 (33%)	0.007**
p.Leu483Pro/p.Asn227Ser	1 (5%)	–	0.593
p.Leu483Pro/p.Asn409Ser	1 (5%)	–	0.593
p.Leu483Pro/p.Phe252Ile	1 (5%)	–	0.593
p.Leu483Pro/p.Arg170His	–	1 (17%)	0.061
p.Leu483Pro/p.Arg159Trp	1 (5%)	1 (17%)	0.335
*RecNci*I/p.Asp448His	1 (5%)	–	0.593
*RecNci*I/p.Arg159Trp	–	–	1.000
p.Phe252Val/p.328Alafs*12	–	1 (17%)	0.061
*Initial presentation*			
Hepatosplenomegaly	21 (100%)	1 (17%)	< 0.001***
Anemia/thrombocytopenia	20 (95%)	1 (17%)	< 0.001***
Bone pain/bone crisis	5 (24%)	0 (0%)	0.194
Abnormal eye movements	2 (10%)	1 (17%)	0.630
Poor feeding	1 (5%)	1 (17%)	0.335
Ichthyosis	0 (0%)	1 (17%)	0.061
Median [IQR] age at diagnosis, years	3.5 [2–24.41]	0.05 [0.05–0.20]	< 0.001***
Median [IQR] age at symptom onset, years	3.5 [2–24.41]	0.5 [0.05–1.37]	0.002**
Median [IQR] age at ERT, years	4.98 [2.18–30.03]	0.86 [0.20–2.43]	0.013*
5-year survival	20 (95%)	3 (50%)	0.007**

The NBS-diagnosed patient with the genotype p.Arg159Trp/p.Leu483Pro patient was unclassified given remaining symptom-free followed by accidental death

IQR, interquartile range

^†^*RecNci*I: gene conversion with *GBAP*. p.Asp448His, p.Leu483Pro, p.Ala495Pro

Statistical differences are shown as *p < 0.05, **p < 0.01, and ***p < 0.001

### Age of diagnosis decreased with years

**Fig. 1:**
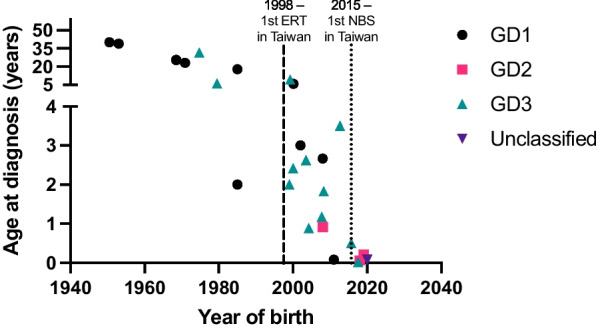
Age at diagnosis across years. The dashed line across 1998 denotes the year when ERT became available for Gaucher disease in Taiwan. The dotted line across 2015 denotes the year that the newborn screening program became available for Gaucher disease in Taiwan

Consistent with the corresponding disease severity, the median ages at diagnosis after clinical suspicion were the earliest for patients with GD2 (0.92 years) and the latest for patients with GD1 (24.41 years, IQR [3.72–35.65]). With further stratification by the year ERT became available (in 1998), the age of diagnosis during the pre-ERT era was 24.41 years [5.22–33.52] as opposed to 1.18 years [0.15–2.83] during the post-ERT era (Additional file 1: Table S1). In contrast, individuals diagnosed following NBS had a median age at diagnosis of 0.08 years [0.05–0.28] a median age at symptom onset of 0.50 years [0.05–1.37]. As shown in Fig. 1, the ages at diagnosis have remained in the neonatal period since the introduction of NBS for GD in 2015, the post-NBS era.

### Initial phenotypes: clinical cases versus NBS cases

**Fig. 2:**
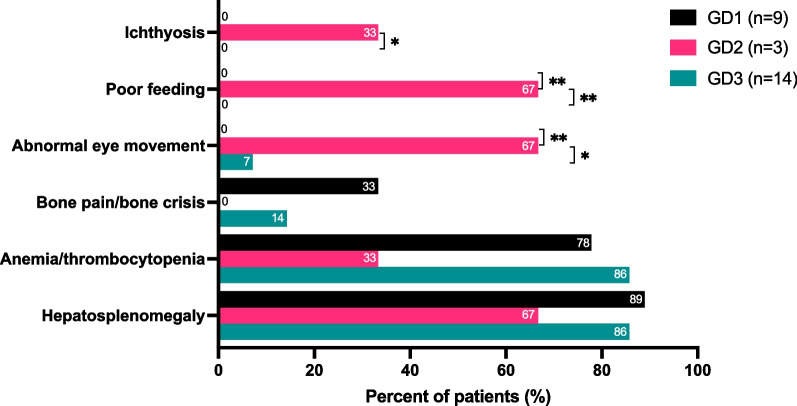
Initial presentations by subtypes. Statistical differences among subtypes are shown as *p < 0.05 and **p < 0.01

Hepatosplenomegaly, anemia, and thrombocytopenia were the main presentations of GD patients (Fig. 2). The degree of splenomegaly was more prominent than that of hepatomegaly prior to treatment (Additional file 2: Figure S1). With stratification by diagnostic method (Table 2), patients diagnosed following NBS were mostly asymptomatic upon diagnosis with statistical significance (*p* < 0.001) observed in analyses of hepatosplenomegaly, anemia, and thrombocytopenia (Additional file 3: Figure S2).

### Management

**Fig. 3:**
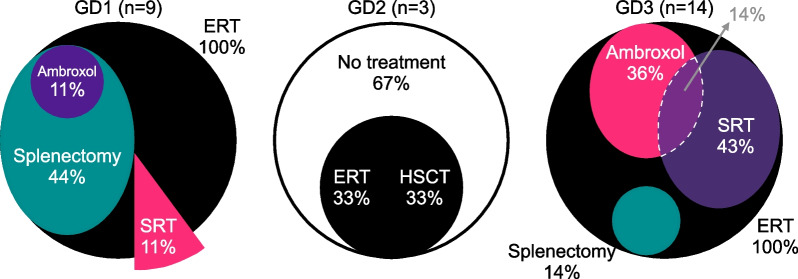
Treatment interventions by subtypes. ERT, enzyme replacement therapy; SRT, substrate reduction therapy; HSCT, hematopoietic stem cell transplantation

ERT was warranted in all patients who were clinically diagnosed (regardless of the timing of diagnosis), except for GD2 patients (Fig. 3). One GD1 patient (11%) was identified as having received ERT and substrate reduction therapy (SRT). The client’s treatment was shifted from ERT, which was complicated by fortnightly intravenous infusions requiring frequent absences from work, to SRT, which involved a once-daily oral regimen on the grounds of the client’s concern about the school-to-work transition in 2021. In addition to this GD1 patient, six GD3 patients (43%) received accompanying SRT owing to protracted neurological manifestations (miglustat only) and/or imaging-proven lymphadenopathies (either miglustat or eliglustat) despite receiving ERT. Ambroxol was used in five GD3 patients (36%) due to the progression of neurological manifestations, including intractable seizures and progressing cognitive decline, as well as one GD1 patient (9%) owing to evolving Parkinson’s disease. Splenectomy was only performed in patients diagnosed during the pre-ERT era (Additional file 4: Figure S3). One NBS-diagnosed patient who underwent ERT in addition to hematopoietic stem cell transplantation (HSCT) was eventually classified as having GD2 (Additional file 5: Figure S4).

### Outcomes

**Fig. 4:**
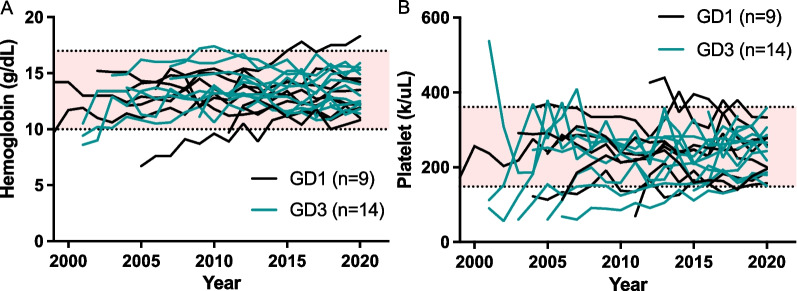
Hematological changes following enzyme replacement therapy (ERT). **A** Changes in hemoglobin levels after ERT. **B** Changes in platelet levels after ERT

**Fig. 5:**
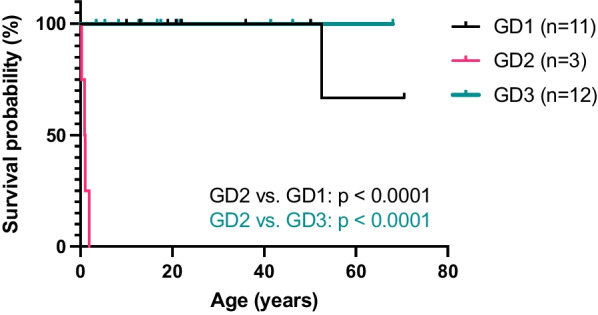
Survival analysis by subtype

In addition to the findings regarding splenectomy, serial changes in the hemoglobin and platelet levels of the GD1 (n = 9) and GD3 (n = 14) patients (Fig. 4) also substantiated the effect of ERT in reversing disease progression, given that anemia and thrombocytopenia have been shown to be indicators of symptom onset. Moreover, the longitudinal transformations revealed that the effect may have been more conspicuous in individuals who were diagnosed by NBS, i.e., after 2015, than in those who were diagnosed clinically, as the NBS-diagnosed individuals convalesced from anemia and thrombocytopenia within a couple of years of treatment, while some of the clinically diagnosed individuals required longer durations. Fifty percent of patients diagnosed during the pre-ERT exhibited various degrees of skeletal manifestations at the time of diagnosis, which was often delayed. In patients diagnosed during the post-ERT era, almost all patients were diagnosed before overt skeletal symptoms appeared (Table S1). The latest assessment age was 19 years [8.3–41.46] (designated by the age of death if the patient was deceased). The duration of follow-up was 14.85 years [4.8–26.88]. While the 5-year survival rates were 0% for GD2 patients and 100% for GD1 and GD3 patients, one GD1 patient died of internal bleeding from a traffic accident at the age of 53 years in late April of 2022 with a follow-up duration of 27 years. The Kaplan–Meier survival curves of the GD1 and GD3 patients both exhibited significant differences (*p* < 0.0001) from that of the GD2 patients (Fig. 5). Of note, one patient who expired by accidental death prior to the onset of any symptom such that subtyping could not be performed, as mentioned in the “Distribution of GD types” section, was excluded from the Kaplan–Meier survival analysis by subtype. Twenty-nine percent (4 of 14) of the GD3 patients had seizures (Table 3). In addition, lymphadenopathies were observed in 50% (7 of 14) of the GD3 patients receiving regular ERT (Additional file 6: Figure S5).

**Table 3:** Genotype, age of onset of symptoms, and current disease phenotypes of GD3 patients

ID	Genotype	Age of onset of symptoms	Oculomotor apraxia	Seizure	Intellectual disability	Lymphadenopathy	Kyphosis
1	p.Leu483Pro/p.Leu483Pro	2.42	+	+	–	+	+
2	p.Leu483Pro/p.Leu483Pro	9.50	+	+	–	–	–
3	p.Leu483Pro/p.Leu483Pro	2.62	–	–	–	+	–
4	p.Leu483Pro/p.Leu483Pro	0.88	–	–	–	+	+
5	p.Leu483Pro/p.Leu483Pro	1.18	+	+	+	+	+
6	p.Leu483Pro/p.Leu483Pro	1.83	–	–	–	+	+
7	p.Leu483Pro/p.Leu483Pro	3.50	+	–	–	+	–
8	p.Leu483Pro/p.Leu483Pro	1.23	+	+	–	+	–
9	p.Leu483Pro/p.Leu483Pro	2.00	–	–	+	–	+
10	p.Leu483Pro/p.Leu483Pro	18	+	–	–	–	–
11	p.Leu483Pro/p.Leu483Pro	2	+	–	–	–	+
12	p.Leu483Pro/p.Asp448His	31.7	+	–	–	–	–
13	p.Leu483Pro/p.Asp448His	6.29	+	–	–	–	+
14	p.Leu483Pro/*RecNci*I^†^	0.10	+	–	+	–	–

^†^*RecNci*I: gene conversion with *GBAP*. p.Asp448His, p.Leu483Pro, p.Ala495Pro

## Discussion

### Study findings

This study is the first cohort study to provide a comparison of the GD population diagnosed during the pre-NBS and post-NBS eras in Taiwan. It is of crucial importance to understand the epidemiology as well as the clinical characteristics when designing a treatment strategy for a rare disease such as GD. In 2006, Wan et al. first reported on the GD cohort in Taiwan via their extensive study of GD disease mutations a year after NBS became available for GD [7]. With sixteen years of cumulation, GD patients diagnosed during the post-NBS era were juxtaposed with those diagnosed during the pre-NBS era. Our cohort encompassed 64% of the Gaucher population in Taiwan, of which 1/7 were diagnosed during the post-NBS era. This study showed that the distribution of subtypes in Taiwan varies greatly from that worldwide. Moreover, the most prevalent genotype worldwide was p.Asp409Ser/p.Asp409Ser (44.2% [374/847]) as opposed to p.Leu483Pro/p.Leu483Pro (41% [11/27]) in our cohort, which coincides with the mutation analysis by Wan et al.; the worldwide prevalence of p.Leu483Pro/p.Leu483Pro was 2.2% (19/847) worldwide based on the Gaucher Outcome Survey analysis by Zimran et al. [7, 29]. However, as only patients visiting NTUH or CMUH between 1998 and 2021 were included, patients who were cared for in other medical institutes or those who died before 1998 were not included in this study.

ERT was effective in ameliorating patients’ clinical symptoms, including visceral, hematological, and skeletal manifestations. With the integration of the NBS program, an increasing number of GD patients are able to receive ERT given the advantage of early diagnosis in providing disease recognition and preventing diagnostic delays. However, as NBS for GD was not available until 2015, ß-glucocerebrosidase enzyme activity should be checked if the clinical suspicion of GD remains strong. Due to the presence of pseudogene of *GBA1* with possibilities of recombination, there may be limitations in diagnostic genotyping, especially in short read next-generation sequencing or Sanger sequencing without prelong-range PCR amplification, which further highlights the importance of enzyme assays.

GD patients have been able to receive regular treatment since 1998 under the coverage of the health care system in Taiwan. With the introduction of NBS for GD in 2015, a distinctive representation of the pre-ERT era, post-ERT clinical era, and post-ERT NBS era (or simply the post-NBS era) is apparent in the Gaucher population. From our data, nearly 90% of the patients in this cohort had either the GD1 (33%) or GD3 (52%) subtype, as opposed to approximately 90% of the patients with the GD1 subtype reported worldwide, highlighting the phenotypic uniqueness of our cohort [10]. While neuro-ophthalmologic study was not performed, patients were assessed at intervals by the treating physicians, who made records of notable interval changes. Records of abnormal eye movements were detailed in medical charts of patients with oculomotor apraxia, and electroencephalographic studies were traceable for patients with clinical suspicion of seizures. Since the introduction of NBS for GD in Taiwan, a redistribution in subtypes has been noted, with an increase in individuals with GD2. To be exact, the diagnoses can be made within the neonatal period, curtailing the symptom-to-treatment interval, which enables these patients to receive early intervention. In contrast with the worldwide distribution of 5% reported for the GD3 subtype [8], it was the most common subtype in our cohort, with a prevalence of 52%. Moreover, patients with GD in Taiwan are entitled to treatment without having to cope with economic hardships.

### Juxtaposition of symptoms in Taiwan and other places around the world

Hepatosplenomegaly, anemia, and thrombocytopenia were the main presentations in clinically diagnosed GD patients across the eras. ERT is approved for the treatment of both GD1 and GD3 patients in Taiwan. For GD2 patients, ERT is sometimes administered for palliative measures to relieve visceral manifestations or is given until the diagnosis of GD3 is disproved [30]. With the use of ERT, patients are exempt from splenectomy, which was the treatment of choice for Gaucher disease before the advent of ERT [31], indicating the effect of ERT in relieving the disease burden. Moreover, most patients display thrombocytopenia and anemia in their initial presentations, with less severity in the GD1 group. Under regular ERT, patients with GD1 and GD3 are able to reinstate hematological stabilizations to impede anemic and thrombocytopenic events, which agrees with the current use of ERT [32]. Although skeletal manifestations affect approximately 50% of patients diagnosed during the pre-ERT era, they were nonexistent in those diagnosed in the post-ERT era. However, while Charrow et al. showed that some skeletal improvement could be attained by ERT, it seemed to be difficult to exercise such an effect for the majority of subjects exhibiting skeletal symptoms [12]. As the recuperation of already established bone disease is difficult or even impossible with ERT, possibly due to altered vascularization, fibrosis, or differing ability of the subpopulations of Gaucher cells in the bone marrow to take up the exogenous enzyme [6, 33, 34], the difference in skeletal manifestations between individuals diagnosed during the pre- and the post-ERT eras reiterates the importance of early diagnosis, which allows early treatment to improve outcomes.

### Lesson from GD2

With NBS, the GD community exhibits a greater proportion of GD2 than previously anticipated. This could be attributed to the rarity of the disease, the phenotypes of which are likely to be unrecognized, let alone precisely diagnosed before disease progression. In our cohort, all except one patient diagnosed by NBS were asymptomatic, which concurs with a previous pilot study on NBS for LSDs [35]. The only symptomatic GD2 patient at the time of diagnosis by NBS initially presented with hepatosplenomegaly, anemia, thrombocytopenia, oculomotor apraxia, and ichthyosis and expired at 3 months of age. NBS helped in rapid diagnostic confirmation. The only GD2 patient who underwent treatment was asymptomatic when diagnosed by NBS with confirmation of the compound heterozygous p.Leu483Pro/*RecNci*I genotype at 2.5 months old. Due to the emergence of hepatosplenomegaly at 5 months of age in addition to squinting, HSCT and ERT were provided promptly in the hope that the patient had the GD3 subtype. While studies comparing the safety and efficacy of HSCT to ERT and SRT are scarce, treatment options with HSCT have been proposed in the context of neuronopathic GD, though the effectiveness is limited [36]. However, as the deterioration progressed, the diagnosis of GD2 was justified, and the patient died at 1 year 11 months of age. Considering the difference in the subtype distributions during the pre-NBS era and post-NBS era, GD patients with severe symptoms were likely to be underdiagnosed during the pre-NBS era.

### Unmet needs of GD3 patients

Patients with GD3 exhibit neurological presentations in addition to visceral, hematological, and skeletal involvement. While the majority of symptoms are amenable to ERT, neurological presentations progress owing to the inability of ERT to cross the blood–brain barrier. As demonstrated by Narita et al. and Aries et al., chaperone therapy with ambroxol has the potential to provide neurological efficacy for both GD3 and GD2 given its ability to cross the blood‒brain barrier as a small molecule [37, 38]. In our cohort, almost half of the GD3 (5 of 12) patients were prescribed ambroxol due to the progression of neurological manifestations. One patient was noted to exhibit improvement of cognitive function [39]. Another patient showed improved development delay and seizure. Another patient who presented with seizure attacks that became refractory after being relatively well controlled with antiepileptic drugs for 7 years was put on ambroxol in addition to 4 antiepileptic medications. Improvement was not noted until after the addition of lacosamide a year later, and mild diffuse brain atrophy was evident under magnetic resonance study. However, another patient exhibited aggravation of neurological symptoms after undergoing spine surgery while receiving ambroxol. The remaining patient was stationary for neurological progression with the addition of ambroxol with a follow-up period of 3.5 years. In addition, severe lymphadenopathies were encountered in GD3 patients, especially those with the homozygous p.Leu483Pro genotype, indicating resistance to soft tissue involvement in GD3 patients receiving ERT [32]. Although eliglustat benefited lymphadenopathies in GD3 patients, it did not have effects on their neurological symptoms [26]. Managing GD3 patients with these neurological presentations remains an unmet need, which accordingly calls for new treatments.

### Circumspection on neuronopathic patients

Although their prognoses vary greatly, the symptomatology of GD2 and GD3 patients may not always be discernible initially, especially for those diagnosed by NBS. The genotype p.Leu483Pro/*RecNci*I was shared by one GD2 patient and one GD3 patient (patient 14 of Table 3). They were both asymptomatic when diagnosed with GD by NBS with low glucocerebrosidase activities of 0.53 μmol/hr/L and 0.47 μmol/hr/L, respectively. ERT was applied in the GD2 patient after abnormal eye movement was observed at the age of 6 months. The neurological progression of the GD2 patient deteriorated despite management with ERT alongside HSCT and he expired after 20 and a half weeks of treatment. On the other hand, ERT was implemented early in the GD3 patient with low platelet count, elevation of lyso-Gb1, and borderline splenomegaly at the age of 5 weeks [39]. Ambroxol was later added for neurological features of mild horizontal gaze palsy and trunk rigidity emerged at 6 months of age. Delayed milestone in gross motor without regression or seizure was noted at 18-months-old. Under active management with ERT and ambroxol, she celebrated her fifth birthday with a stabilized neurological progression. At 4 years 3 months, the WISC result for Full-scale IQ (FIQ) was 71 with a Verbal Comprehension Index (VCI) of 93. She was able to walk independently, run, and jump with ataxia, hypertonicity and gaze palsy. As per her current presentation, the clinical classification was GD3 although the genotype is p.Leu483Pro/*RecNci*I. The wide spectrum of the same genotype recapitulates not only the importance of early diagnosis to allow timely intervention but also the significance of not withholding ERT and/or ambroxol on patients with genotypes of unfavorable outcome forecast.

## Limitations of the study

There are still limitations to this study. First, the number of patients in our cohort was a small fraction of the world total. Second, patients cared for in other medical institutes or those who died before 1998, which was highly likely for those with GD2, were not included in this study. Such a limitation is evident in that the mutation analysis in 2001 revealed five GD2 patients in Taiwan [40]. Furthermore, some clinical data were from so long ago that neither written nor electronic information could be fully retrieved. Last, initial biomarkers were not available in old cases. In addition, the clinical phenotype is a spectrum; sometimes, it is difficult to clearly distinguish subtypes, especially when the course of disease is slow. For example, the majority of Leu483Pro homozygous patients presented as having GD3. However, patients 10 and 11 of Table 3, both aged 37 currently, only manifest very subtle gaze palsy for the classification of GD3. Since the clinical manifestations of GD patients may change with time, we always keep in mind the importance of re-evaluation and reclassification in these patients.

## Conclusion

The Gaucher population in Taiwan exhibits phenotypic uniqueness, such that GD3 was the major subtype. Furthermore, the NBS program is able to advance the age at diagnosis such that better outcomes may be achieved with early intervention. Moreover, NBS prevents delayed diagnosis and precludes at-risk families from bearing another affected child resulting from untimely implementation of genetic counseling and prenatal diagnosis. However, while ERT is able to reverse hepatosplenomegaly and hematological complications, more treatment options are needed for the management of other symptoms.

### Supplementary Information

**Additional file 1. Table S1**. Patient subdivision according to year of birth before and after ERT introduction in Taiwan (1998).

**Additional file 2. Figure S1**. Liver and spleen sizes. Statistical difference among eras is shown as *p < 0.05.

**Additional file 3. Figure S2**. Comparison of initial presentation among eras. Statistical differences between eras are shown as *p < 0.05, **p < 0.01, and ***p < 0.001. HSCT, hematopoietic stem cell transplantation; SRT, substrate reduction therapy; ERT, enzyme replacement therapy.

**Additional file 4. Figure S3**. Interventions by eras. Statistical differences among eras are shown as *p < 0.05, **p < 0.01, and ***p < 0.001. HSCT, hematopoietic stem cell transplantation; SRT, substrate reduction therapy; ERT, enzyme replacement therapy.

**Additional file 5. Figure S4**. Initial hematological presentation–hemoglobin levels.

**Additional file 6. Figure S5**. Initial hematological presentation—platelet levels.

## Data Availability

The data are not publicly available given the sensitive nature of identifiable human data. Reasonable requests can be made to the corresponding author via this email: ncleentu@ntu.edu.tw.
