# Supplementary material for: Changing clinical manifestations of Gaucher disease in Taiwan
Source: Orphanet J Rare Dis. 2023 Sep 15;18:293. doi: 10.1186/s13023-023-02895-z (PMC10502973; doi:10.1186/s13023-023-02895-z)
Supplement: Supplementary file 1 — Additional file 1. Table S1. Patient subdivision according to year of birth before and after ERT introduction in Taiwan (1998). [file 13023_2023_2895_MOESM1_ESM.docx]

**Supplementary**

Table S1. Patient subdivision according to year of birth before and after ERT introduction in Taiwan (1998)

|  | Before 1998 | After 1998 | | | | |
| --- | --- | --- | --- | --- | --- | --- |
|  |  | Total | *p* value | Clinical | NBS | *p* value |
| Number of patients (n=27) | 10 (37%) | 17 (63%) |  | 11 (65%) | 6 (35%) |  |
| Sex |  |  | 0.662 |  |  | 0.638 |
| Male | 5 (50%) | 10 (59%) |  | 6 (55%) | 4 (67%) |  |
| Female | 5 (50%) | 7 (41%) |  | 5 (45%) | 2 (33%) |  |
| Subtype |  |  |  |  |  |  |
| GD1 | 5 (50%) | 4 (24%) | 0.167 | 3 (27%) | 1 (17%) | 0.633 |
| GD2 | 0 (0%) | 3 (18%) | 0.167 | 1 (9%) | 2 (33%) | 0.224 |
| GD3 | 5 (50%) | 9 (53%) | 0.885 | 7 (64%) | 2 (33%) | 0.246 |
| Unclassified | 0 (0%) | 1 (6%) | 0.443 | 0 (0%) | 1 (17%) | 0.176 |
| Molecular diagnosis |  |  |  |  |  |  |
| p.Leu483Pro homozygous | 3 (30%) | 8 (47%) | 0.393 | 7 (64%) | 1 (17%) | 0.072 |
| p.Leu483Pro/*RecNci*I | 0 (0%) | 2 (12%) | 0.269 | 0 (0%) | 2 (33%) | 0.048* |
| p.Leu483Pro heterozygous | 7 (70%) | 4 (24%) | 0.020* | 2 (18%) | 2 (33%) | 0.495 |
| *RecNci*I heterozygous | 0 (0%) | 2 (12%) | 0.269 | 2 (18%) | 0 (0%) | 0.281 |
| p.Phe252Val/p.328Alafs*12 | 0 (0%) | 1 (6%) | 0.443 | 0 (0%) | 1 (17%) | 0.176 |
| Medan [IQR] age at diagnosis, years | 24.41 [5.22–33.52] | 1.18 [0.15–2.83] | <0.001*** | 2.62 [1.18–3.50] | 0.08 [0.05–0.28] | <0.001*** |
| Presentations |  |  |  |  |  |  |
| Hepatosplenomegaly | 10 (100%) | 12 (71%) | 0.062 | 11 (100%) | 1 (17%) | <0.001*** |
| Anemia/thrombocytopenia | 9 (90%) | 11 (65%) | 0.155 | 10 (91%) | 1 (17%) | 0.003** |
| Bone pain/bone crisis | 5 (50%) | 0 (0%) | 0.002** | 0 (0%) | 0 (0%) | 1.000 |
| Abnormal eye movements | 0 (0%) | 3 (18%) | 0.167 | 2 (18%) | 1 (17%) | 0.939 |
| Poor feeding | 0 (0%) | 2 (12%) | 0.269 | 1 (9%) | 1 (17%) | 0.653 |
| Ichthyosis | 0 (0%) | 1 (6%) | 0.443 | 0 (0%) | 1 (17%) | 0.176 |
| Note: The NBS-diagnosed patient with the genotype p.Arg159Trp/p.Leu483Pro patient was unclassified given remaining symptom-free followed by accidental death.  †*RecNci*I: gene conversion with *GBAP*. p.Asp448His, p.Leu483Pro, p.Ala495Pro.  Statistical differences are shown as **p* <0.05, ***p* <0.01, and ****p* <0.001. | | | | | | |

**Figure legends**

**Figure S1**. Comparison of initial presentations among eras. Statistical differences between eras are shown as **p* <0.05, ***p* <0.01, and ****p* <0.001. HSCT, hematopoietic stem cell transplantation; SRT, substrate reduction therapy; ERT, enzyme replacement therapy.

**Figure S2**. Liver and spleen sizes. Statistical difference among eras is shown as **p* <0.05.

**Figure S3**. Interventions by eras. Statistical differences among eras are shown as **p* <0.05, ***p* <0.01, and ****p* <0.001. HSCT, hematopoietic stem cell transplantation; SRT, substrate reduction therapy; ERT, enzyme replacement therapy.

**Figure S4**. Initial hematological presentation – hemoglobin levels.

**Figure S5**. Initial hematological presentation – platelet levels.
