# Supplementary figures and images for: Changing clinical manifestations of Gaucher disease in Taiwan
Source: Orphanet J Rare Dis. 2023 Sep 15;18:293. doi: 10.1186/s13023-023-02895-z (PMC10502973; doi:10.1186/s13023-023-02895-z)

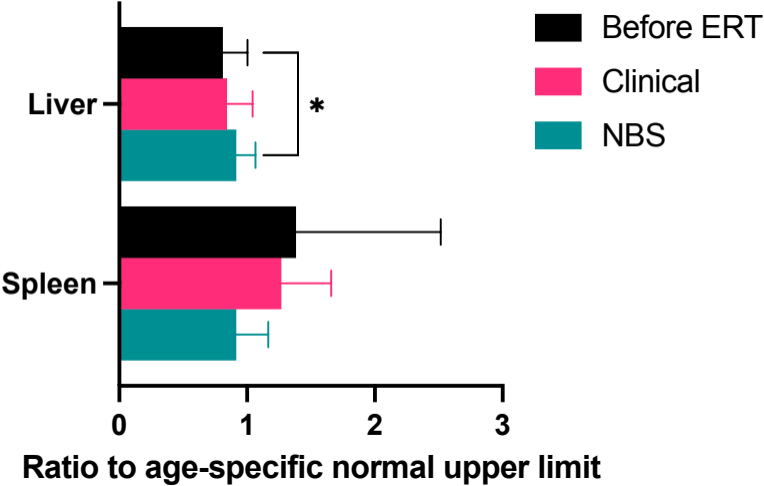

Supplement: Supplementary file 2 — Additional file 2. Figure S1. Liver and spleen sizes. Statistical difference among eras is shown as *p < 0.05. [file 13023_2023_2895_MOESM2_ESM.pdf]

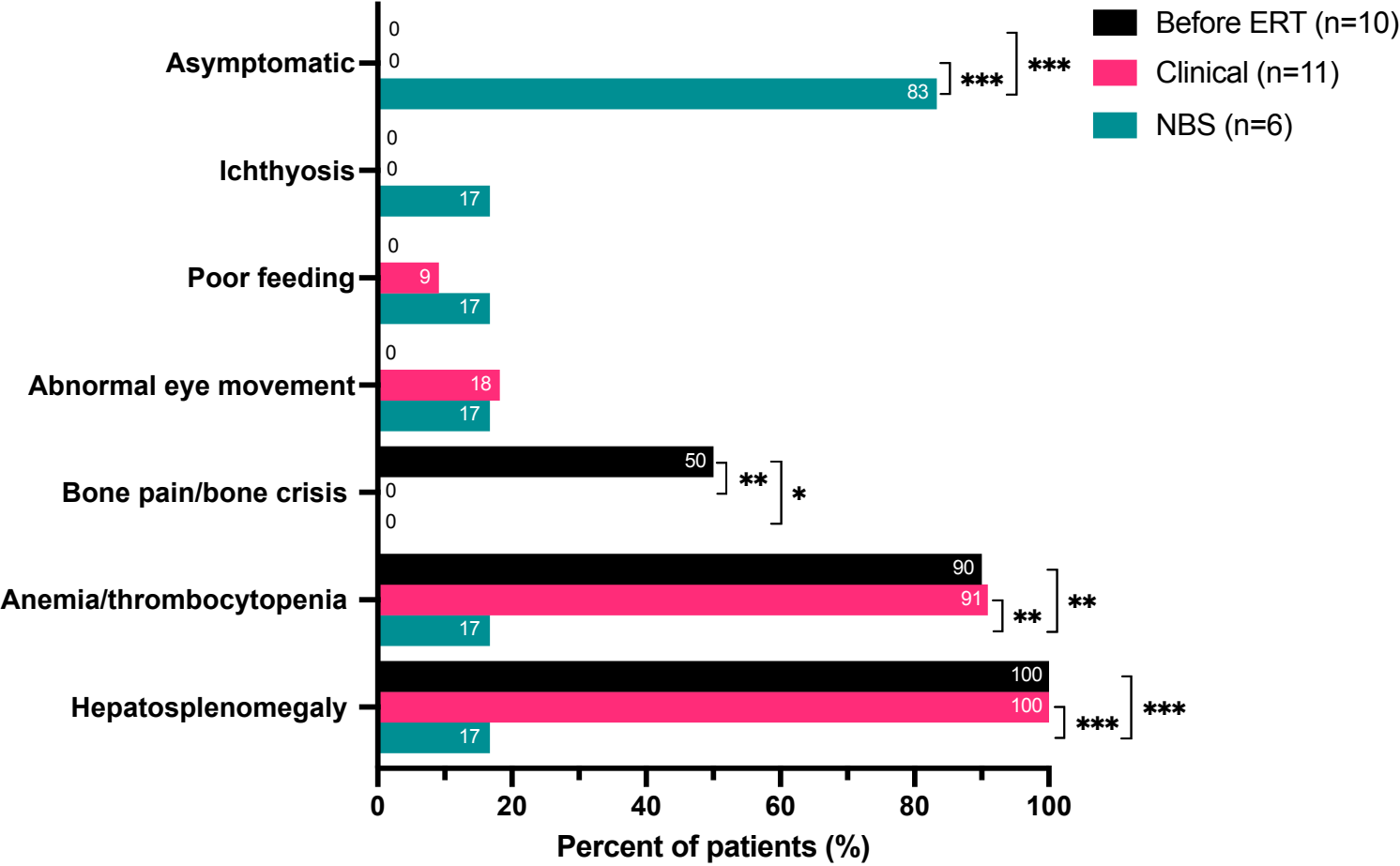

Supplement: Supplementary file 3 — Additional file 3. Figure S2. Comparison of initial presentation among eras. Statistical differences between eras are shown as *p < 0.05, **p < 0.01, and ***p < 0.001. HSCT, hematopoietic stem cell transplantation; SRT, substrate reduction therapy; ERT, enzyme replacement therapy. [file 13023_2023_2895_MOESM3_ESM.pdf]

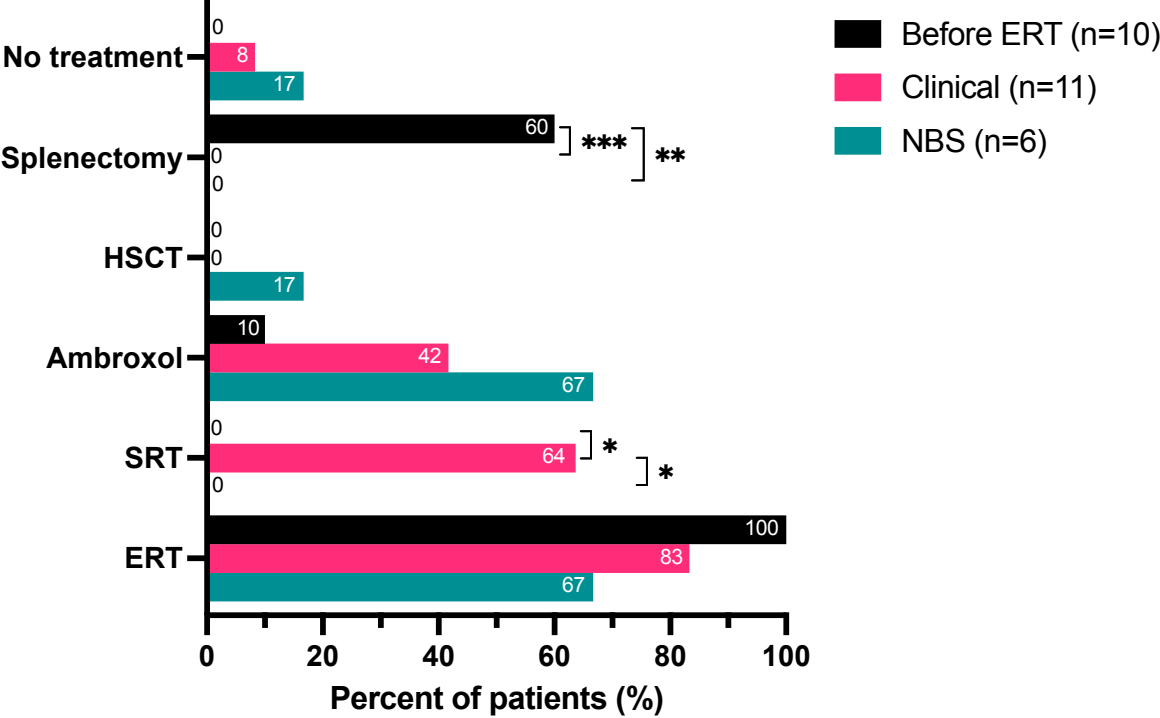

Supplement: Supplementary file 4 — Additional file 4. Figure S3. Interventions by eras. Statistical differences among eras are shown as *p < 0.05, **p < 0.01, and ***p < 0.001. HSCT, hematopoietic stem cell transplantation; SRT, substrate reduction therapy; ERT, enzyme replacement therapy. [file 13023_2023_2895_MOESM4_ESM.pdf]

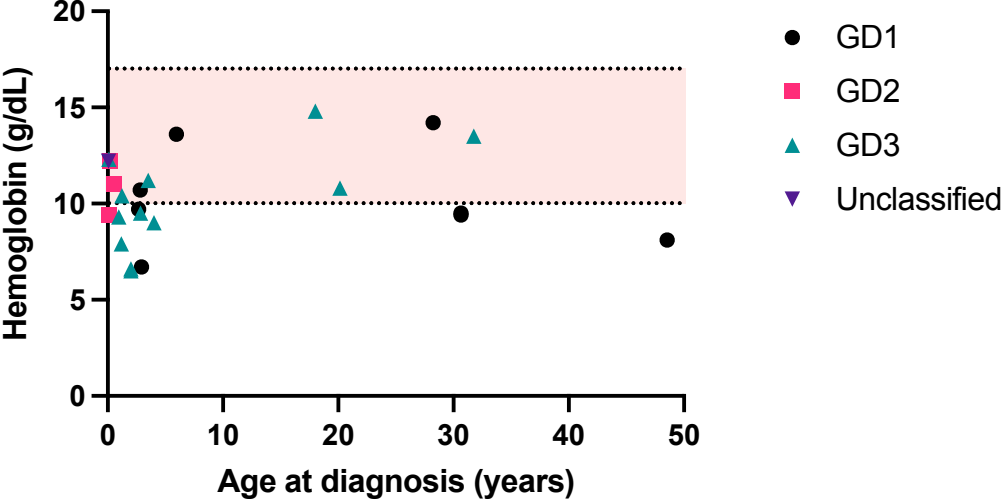

Supplement: Supplementary file 5 — Additional file 5. Figure S4. Initial hematological presentation–hemoglobin levels. [file 13023_2023_2895_MOESM5_ESM.pdf]

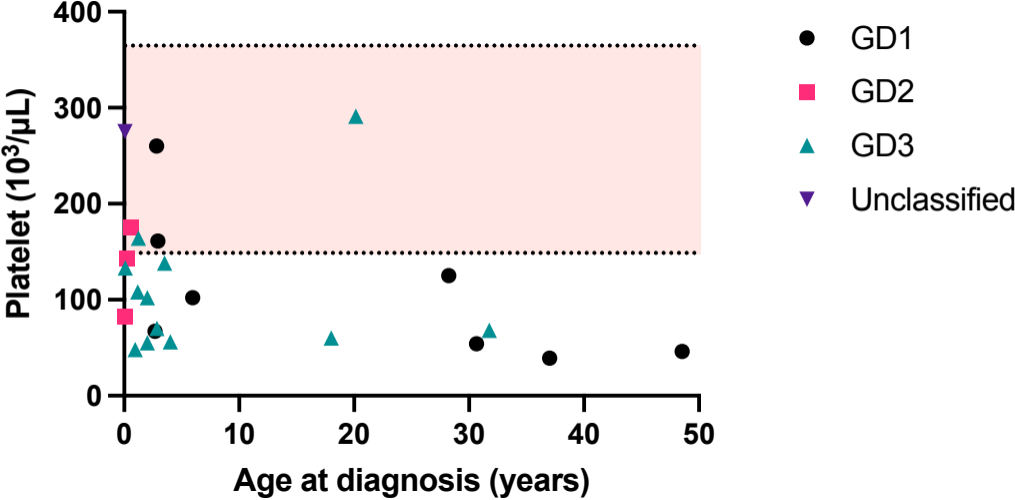

Supplement: Supplementary file 6 — Additional file 6. Figure S5. Initial hematological presentation—platelet levels. [file 13023_2023_2895_MOESM6_ESM.pdf]
